# Relationship between internet use intensity and quality of life in chronic patients during the COVID-19 pandemic: The role of physical exercise and health insurance

**DOI:** 10.3389/fpubh.2022.947465

**Published:** 2022-09-16

**Authors:** Yangyang Wang, Jian Xu, Tian Xie

**Affiliations:** ^1^China Institute for Urban Governance, Shanghai Jiao Tong University, Shanghai, China; ^2^School of International and Public Affairs, Shanghai Jiao Tong University, Shanghai, China; ^3^School of Media and Communication, Shanghai Jiao Tong University, Shanghai, China

**Keywords:** COVID-19 pandemic, internet use intensity, quality of life, health insurance, physical exercise, health management of rural and urban governance

## Abstract

The internet use intensity of human has increased substantially during the COVID-19 Pandemic, and it is severely impacting the well-being of chronic patients. This study aimed to explore the underlying mechanism of the relationship between internet use intensity and quality of life in chronic patients, based on the cross-sectional data from China Family Panel Studies (CFPS) during the COVID-19 Pandemic in 2020. The results showed that the internet use intensity had significant positive association with quality of life among chronic patients, and such association has been found in both urban and rural samples. Among the relationship of internet use intensity and quality of life in chronic patients, the mediating effect of physical exercise reached 10.25%. Furthermore, health insurance positively moderated this relationship. There are new insights for policy recommendations and clinical guidance on the role of physical activity and health insurance aimed at improving chronic patients' quality of life. Meanwhile, in both rural and urban governance, public health agencies should promote the “Internet + Healthcare” program to improve health insurance and physical activity literacy, thus providing a higher level of quality of life for patients with chronic diseases during the COVID-19 Pandemic.

## Introduction

Since the outbreak of the COVID-19 Pandemic in China at the end of 2019, the pandemic has become a global public health event with a continuous impact on social development and people's daily life and health (1, 2). Numerous studies have pointed out that people experienced significantly lower quality of life and more mental health issues during the COVID-19 pandemic (3–5). As a vulnerable group, the quality of life of patients with chronic diseases deserves special attention. Currently, studies show that chronic diseases which contain cardiovascular diseases, cancer, chronic respiratory diseases, and diabetes are the largest cause of death globally (6, 7). As the population aging dramatically, the number of patients with chronic diseases substantially increases in China, and China faces a higher mortality rate than developed countries (8, 9). The previous study showed that chronic patients reported lower levels of quality of life during the COVID-19 Pandemic (10), such as drop-in emotional functioning and social functioning, and severe psychological problems (11). Therefore, it's essential to explore the factors influencing quality of life among chronic patients.

According to the World Health Organization (WHO), quality of life refers to “an individual's perception of their position in the life in the context of the culture in which they live and in relation to their goals, expectations, standards, and concerns” (12). The appraisal of quality of life is subjective, however, it is also affected by many objective factors, among which internet use is a significant factor. The use of digital technology has increased dramatically after the outbreak of COVID-19, up to 90% of the Chinese participants reported longer screen time for study, work, and entertainment (13–15). Many studies have shown that internet use was positively associated with quality of life from different aspects, such as decreasing loneliness (16), improving social relationships and personal well-being (17), and enhancing physical and mental health (18, 19). A study showed that moderate amounts of time spent on online activities are beneficial for enhancing the level of quality of life (19). However, some studies hold different views. The prevalence of internet addiction among vulnerable people in China has increased during the pandemic (20). While more time spent on the internet might increase social isolation and loss of contact with the social environment (21). This means that excessive use of the internet has a negative effect on the quality of life (22, 23). Thus, the relationship between internet use intensity and quality of life is still unclear, and its underlying mechanism is still to be confirmed during COVID-19.

Although a large number of studies have shown that internet use intensity was associated with quality of life, few studies have explored its underlying mediating mechanisms. One study pointed out that physical exercise mediated the relationship between internet use and mental health (24). The internet provides a favorable tool for physical exercises, such as searching for exercise information, using exercise apps (25), providing exercise guidance (26), self-monitoring (27), and transferring exercise data (28). Physical exercise has long been used as a means of rehabilitation for chronic patients. Some study have shown that internet-based physical activity is an effective way for quality of life improvement (29). Meanwhile, physical exercise has a positive effect on the quality of life among patients with chronic conditions, such as chronic brain disorders, chronic liver disease, and type 2 diabetes (30–32). As a significant intervention for patients with chronic diseases, physical exercise plays an irreplaceable role in optimizing bodily functioning (33), reducing morbidity and mortality (34), and improving patients' quality of life. In conclusion, internet use intensity is positively associated with physical exercise, and physical exercise predicts quality of life. Therefore, it can be assumed that physical exercise plays a mediating role in the relationship between internet use intensity and quality of life.

Health insurance has not to be sufficiently considered as a potential moderator for quality of life. The benefits of health insurance improved health-related outcomes in chronic patients (35). Ronksley et al. demonstrate that chronic conditions and distress were significantly related to unsatisfied healthcare needs (36). At the same time, the inclusion of chronic disease drugs in medical insurance reimbursement will benefit patients (37). With the promotion of China's “Internet +Healthcare” program, the internet provides a convenient way for medical consultation, treatment, and health insurance reimbursement. One study pointed that the higher the frequency of internet usage, the more likely Chinese households are to participate in private insurance (38). And patients who had private reimbursement insurance reported a higher quality of life than those with public insurance (39). Besides, some studies revealed that individuals who have health insurance reported a higher quality of life than those with no health insurance (40, 41). Hence, health insurance may moderate the association between internet use intensity and quality of life.

**Figure 1:**
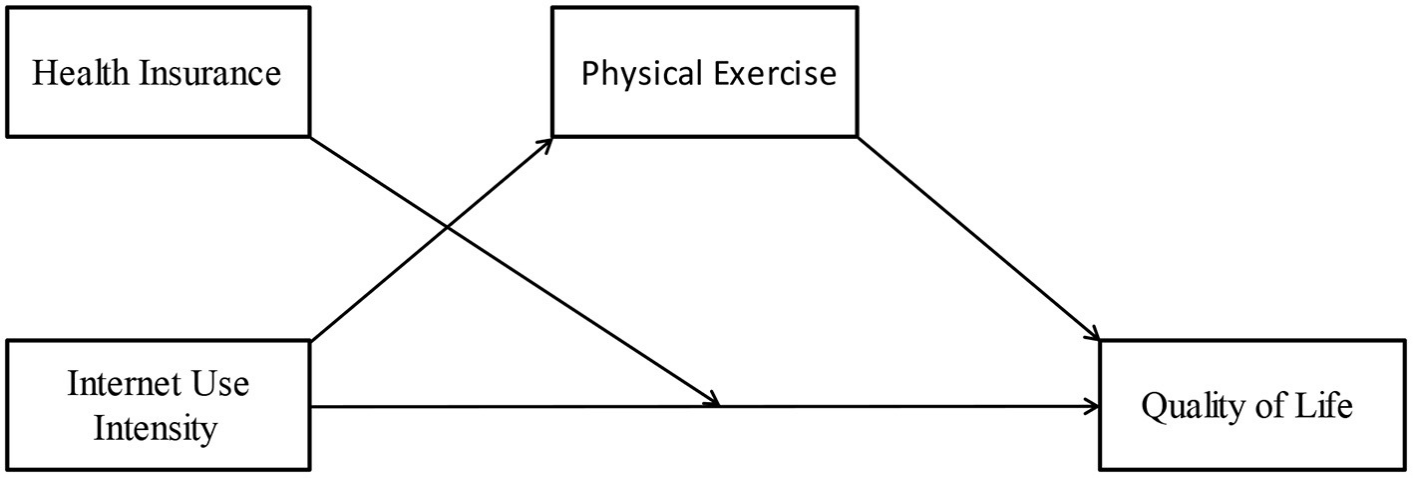
The relationship among internet use intensity, quality of life, physical exercise, and health insurance.

Taken together, the main goal of the current study is to explore the impact mechanism of internet use intensity on the quality of life of chronic patients during the COVID-19 pandemic. In order to improve the quality of life of chronic patients, policy recommendations and clinical guidance will be provided. There were the following hypotheses: (H1) There will be a positive relationship between internet use intensity and quality of life. (H2) Physical exercise mediates the relationship between internet use intensity and quality of life. (H3) Health insurance plays a moderating role in the relationship between internet use intensity and quality of life (see Figure 1).

## Methods

### Data and participants

The current study obtains the dataset from China Family Panel Studies (CFPS), funded by Peking University and carried out by the Institute of Social Science Survey of Peking University. It covers 25 provinces/municipalities/autonomous regions, and was officially launched in 2010 with a target sample size of 16,000 households. The CFPS sample is a multi-stage probability sample drawn using the implicit stratification method, and each subsample is drawn in three stages. The first two stages of the sampling process use official administrative division information. This is because the administrative division structure of mainland China is strictly hierarchical and covers the entire population of mainland China. The third stage was to create end sampling frames in the selected sample villages/residences using the list of households obtained from the village survey map, and to draw a sample of households by expanding the sample size according to circular equidistant sampling with a random starting point to ensure that each sample village/residences could fulfill the target of 25 households. The 2020 wave of CFPS was conducted from July to December 2020 during the COVID-19 Pandemic and contained 28,590 individuals. The target population of this study was respondents with chronic diseases, and a final sample of 3,313 cases with chronic diseases was used after screening and cleaning invalid samples. Among them, 1,777 (53.64%) were female and 1,536 (46.36%) were male; 1,582 (47.75%) were rural residents while 1,731 (52.25%) were urban residents.

### Variables

#### Dependent variable

Quality of Life. Since Flanagan developed the Quality of Life Scale (QOLS) in 1978 (42), measuring quality of life in terms of five dimensions has been accepted in many studies (43–45). The five dimensions of the QOLS include physical and material well-being; relationships with other people; social, community and civic activities; personal development and fulfillment; and recreation. Based on these five dimensions, this study selected 14 approximate question items to measure the quality of life from CFPS 2020, namely: (1) have health insurance (From the question “What health insurance do you have?”); (2) have high local income (From the question “How would you rate your income in your local area?”); (3) have good health (From the question “How do you consider yourself to be in good health?”); (4) have good interpersonal relationships (From “How well-connected do you think you are?”); (5) have a sense of subjective well-being (From the question “How happy do you think you are?”); (6) post comments related to political issues and national events (From the question “In the past 12 months, have you made any statements on your website related to political issues and national issues?”); (7) vote in village/neighborhood council elections (From the question “In the last 5 years, have you voted in village/neighborhood council elections?”); (8) read books in the past year (From the question “In the past 12 months, excluding reading for work and exams, have you read any books?”); (9) have high life satisfaction (From the question “How satisfied do you rate yourself with your life? “); (10) have confidence in the future (From the question “What is your level of confidence in your future?”); (11) think the living standard is likely to improve (From the question “In today's society, there are still many opportunities for people like me to improve their living standards”); (12) play online games (From the question “In the past week, have you played online games?”); (13) spend time watching TV, movies and other video programs (From the question “In general, how many hours per week do you spend watching TV, movies and other video programs by any means?”); (14) share my work or life in social media frequently (From the question “In the past year, how often did you share your work or life in your WeChat Friend Circle?”). For each of the above question items, respondents were coded “1” if they exceeded the median of the sample, otherwise they were coded “0.” Finally, the coded answers of the 14 questions were summed to obtain a score between 0 and 14, with higher values indicating a higher quality of life.

#### Independent variable

Internet Use Intensity. According to the related studies (46–49), time spent online is measured by two questions: “In general, how long do you spend online with your mobile devices each day?” and “In general, how long do you spend online with the internet each day?”. The total time spent online using computers and mobile devices was summed by hours per day to measure the internet use intensity of the respondents. The more time spent on the internet per day, the higher the internet use intensity of the respondents.

#### Mediating variable

Physical Exercise. Physical activity was measured with a single-item question that asked “How often did you participate in physical fitness and leisure activities in the past 12 months?” that ranged from 1 (never participate) to 8 (twice a day or more). The higher the coded value, the more frequent the physical activity participation.

#### Moderating variable

Health Insurance. In CFPS 2020, respondents were asked about their health insurance coverage by one question “What health insurance coverage do you have?”. According to the respondent's participation in health insurance, “have at least one type of health insurance” was coded as “1”; “none of health insurance” was coded as “0.”

#### Control variables

Based on previous studies (50–52), socio-demographic characteristics, residential status, health behavior, and economic status were selected as control variables. Socio-demographic characteristics included gender (male, female), age, marital status (unmarried, other), and educational experience (code “not educated” as “1,” “primary school” as “2,” “junior high school” as “3,” “high School/junior high school/technical school/vocational high school” as “4,” and “college and above” as “5”; higher coding value indicates higher education level). Residence status includes residence type (urban, rural). Health behaviors include smoking, drinking, and self-assessed health status (Likert five-degree scale; the higher the number, the healthier it is). Economic status included annual personal income (unit is 100,000 RMB) and annual medical costs (include total hospital expenses and other injury expenses; unit is 100,000 RMB).

### Statistical analysis

Descriptive analyses were conducted for all variables. The effect of internet use intensity on quality of life was analyzed using the ordered logistic regression model (Model 1) and separately for the urban subsample (Model 2) and the rural subsample (Model 3). The mediating effect of physical exercise was examined using the macro PROCESS4.0 tool (53) (Model 4, Model 5, Model 6). The moderating effect of health insurance was examined by constructing an interaction of health insurance × internet use intensity via ordered logistic regression model (Model 7). Robustness tests were conducted by recoding the independent and dependent variables and selecting urban and rural subsamples using OLS (Model 8, Model 9, Model 10). All the above analyses were performed with the help of SPSS26 and Stata17.

## Results

### Descriptive statistics of all variables

Table 1 presents the descriptive statistics for all variables. The mean value of quality of life is 6.08 (SD, 2.03), indicating that the respondents' quality of life is low on the average, and many respondents spend close to the average amount of time using the internet. the mean value of internet use intensity is 1.41(SD, 2.80) hours per day, indicating that most of the respondents spend very little time using the internet, and only a few users spend a lot of time using the internet, with great variation between respondents. The mean value of physical exercise is 2.89 (SD, 2.60), indicating that most respondents have very little time doing physical exercise. And the mean value for health insurance was 0.93 (SD, 0.26), indicating that the majority of respondents had at least one type of insurance. In addition, the male and female samples were approximately equal in proportion, with an average age of 56.44; most samples were married, and had a low overall education level; more samples live in urban than rural but only a small number of people smoking and drinking; poor self-assessed health status, and low average annual personal income and average annual medical costs.

**Table 1:** Descriptive statistics of all variables (*N* = 3,313).

**Variables**	**Type of statistics**	**Full sample**
Quality of life	Mean (SD)	6.08 (2.03)
Internet use intensity	Mean (SD)	1.41 (2.80)
Physical exercise	Mean (SD)	2.89 (2.60)
Health insurance	Mean (SD)	0.93 (0.26)
Gender	*N* (%)	
Female		1,777 (53.64%)
Male		1,536 (46.36%)
Age	Mean (SD)	56.44 (14.34)
Marriage status	*N* (%)	
Married		3,151 (95.11%)
Others		162 (4.89%)
Education level	*N* (%)	
Not educated		2,688 (81.13%)
Primary school		80 (2.41%)
Junior high school		198 (5.98%)
HJTV		248 (7.49%)
College and above		99 (2.99%)
Residence type	*N* (%)	
Rural		1,582 (47.75%)
Urban		1,731 (52.25%)
Smoking	*N* (%)	
No		2,519 (76.03%)
Yes		794 (23.97%)
Drinking	*N* (%)	
No		2,951 (89.07%)
Yes		362 (10.93%)
Self-assessed health status	Mean (SD)	2.11 (1.12)
Annual personal income	Mean (SD)	0.13 (0.34)
Annual medical costs	Mean (SD)	0.10 (0.28)

HJTV, High School/Junior High School/Technical School/Vocational High School.

### Correlation analysis between main variables

Table 2 shows the results of the bivariate correlation analysis using Pearson correlation for the main variables. Quality of life and internet use intensity (r = 0.099, *p* < 0.001), health insurance (r = 0.172, *p* < 0.001) and physical exercise (r = 0.149, *p* < 0.001) were significantly and positively correlated. It indicates that respondents with higher intensity of internet use, having health insurance and more frequent physical exercise have a higher quality of life. Physical exercise and internet use intensity (r = 0.112, *p* < 0.001) and health insurance (r = 0.047, *p* < 0.001) were significantly positively correlated. This suggests that respondents with more physical exercise and higher internet use intensity are more likely to have health insurance, but there is no association between internet use intensity and health insurance.

**Table 2:** Correlation analysis between main variables (*N* = 3,313).

**Variables**	**Quality of life**	**Internet use intensity**	**Health insurance**	**Physical exercise**
Quality of life	–	–	–	–
Internet use intensity	0.099^***^	–	–	–
Health insurance	0.172^***^	0.002	–	–
Physical exercise	0.149^***^	0.112^***^	0.047^***^	–

*** p < 0.001.

### Internet use intensity and quality of life

In this part of the analysis, three ordered logistic regression models were constructed separately to examine the impact of internet use intensity on quality of life. Considering the large gap between rural and urban areas in China, this effect still presents in terms of internet use. Separate regression models were constructed for the rural sample and the urban sample. The total sample size is 3,313, 1,582 for the rural sample, and 1,731 for the urban sample.

Table 3 shows the regression results of internet use intensity on quality of life after excluding the effect of multicollinearity. The odds ratios (OR) of the ordered logistic regression model are reported here. The results indicate that there is a significant positive effect of internet use intensity on quality of life among respondents with chronic diseases, supporting hypothesis H1. The full sample regression of model 1 indicates that the OR of internet use intensity on quality of life is 1.078, which is statistically significant at the 1% level of significance. By increasing the internet use intensity of respondents with chronic diseases by 1 h per day, it improves the quality of life by 7.8%, holding other control variables constant. This reveals that the stronger the internet use intensity, the greater the probability of improving the quality of life for respondents with chronic diseases. For model 2, the regression results for the rural subsample show that the OR of the effect of internet use intensity on quality of life is 1.138, which is statistically significant at the 1% level of significance. This suggests that a 1 h increase in daily internet use intensity among respondents with chronic diseases in rural areas can bring about a 13.8% improvement in quality of life, with other control variables held constant. In model 3, the regression results for the urban subsample show that the OR of internet use intensity on quality of life is 1.059, which is statistically significant at the 1% level of significance. This indicates that an increase in internet use intensity of 1 h per day among chronic disease respondents in urban areas can lead to a 5.9% improvement in quality of life, when keeping other control variables constant.

**Table 3:** Results of global and subsample regression analysis (*N* = 3,313).

**Variables**	**Full sample**	**Rural sample**	**Urban sample**
	**Model 1**	**Model 2**	**Model 3**
Internet use intensity	1.078^***^	1.138^***^	1.059^***^
	(0.018)	(0.038)	(0.020)
Gender (ref: female)	1.194^**^	1.234^*^	1.163
	(0.094)	(0.141)	(0.125)
Age	1.035^***^	1.039^***^	1.030^***^
	(0.004)	(0.005)	(0.005)
Marriage status (ref: married)	1.254	1.422	1.125
	(0.229)	(0.409)	(0.268)
Education level (ref: Not educated)			
Primary school	1.017	1.416	0.675
	(0.215)	(0.410)	(0.217)
Junior high school	1.428^**^	1.357	1.393
	(0.230)	(0.344)	(0.300)
HJTV	1.917^***^	1.903^**^	1.828^***^
	(0.325)	(0.579)	(0.380)
College and above	1.506^*^	1.536	1.468
	(0.346)	(0.818)	(0.380)
Residence type (ref: rural)	1.087		
	(0.070)		
Health insurance (ref: none)	3.338^***^	3.611^***^	3.130^***^
	(0.416)	(0.706)	(0.497)
Smoking (ref: no)	0.924	1.010	0.833
	(0.084)	(0.133)	(0.106)
Drinking (ref: no)	1.157	1.098	1.207
	(0.125)	(0.171)	(0.183)
Self-assessed health status	1.575^***^	1.635^***^	1.516^***^
	(0.048)	(0.069)	(0.067)
Annual personal income	1.289^*^	1.079	1.388^*^
	(0.184)	(0.222)	(0.233)
Annual medical costs	0.987	0.816	1.072
	(0.107)	(0.173)	(0.158)
Observations	3,313	1,582	1,731
Wald chi^2^	471.05	274.52	205.51
Pseudo *R*^2^	0.037	0.043	0.032
Log pseudolikelihood	−6743.92	−3177.93	−3553.09

The parameters reported are the odds ratios (OR) of the ordered logistic regression model; robust standard errors in parentheses. HJTV indicates High School/Junior High School/Technical School/Vocational High School.

*** p < 0.01,

** p < 0.05,

* p < 0.1.

It also can be found that the positive effect of internet use intensity on quality of life is strongest in the rural sample, second strongest in the overall sample, and weakest in the urban sample. The reason for this difference is that since the COVID-19 Pandemic, the number of new rural internet users far exceeds that of urban internet users (54, 55), making this effect relatively stronger. In addition, age, education level, health insurance, and self-assessed health status all had significant positive effects on quality of life.

### The mediating effect test of physical exercise

Table 4 shows that physical exercise plays a mediating effect in the process of internet use intensity influencing quality of life after excluding the effect of multicollinearity. Specifically, model 4 shows that internet use intensity significantly and positively affects physical exercise (β = 0.104, *p* < 0.01); model 5 suggests that internet use intensity significantly and positively affects quality of life (β = 0.078, *p* < 0.01); model 6 indicates that internet use intensity (β = 0.07, *p* < 0.01) and physical exercise (β = 0.08, *p* < 0.01) significantly and positively affected quality of life. It suggests that physical exercise mediates the relationship between internet use intensity and quality of life. And hypothesis H2 was supported. Moreover, the physical exercise here exerts a partial mediating effect.

**Table 4:** Results of the mediating effect test (*N* = 3,313).

**Variables/Dependent variable**	**Physical exercise**	**Quality of life**	**Quality of life**
	**Model 4**	**Model 5**	**Model 6**
Internet use intensity	0.104^***^	0.078^***^	0.070^***^
	(0.02)	(0.015)	(0.015)
Physical exercise			0.08^***^
			(0.013)
Gender (ref: female)	0.326^**^	0.201^**^	0.176^**^
	(0.108)	(0.08)	(0.08)
Age	0.017^**^	0.037^***^	0.036
	(0.005)	(0.004)	
Marriage status (ref: married)	0.306	0.289^*^	0.265
	(0.224)	(0.168)	(0.167)
Education level (ref: Not educated)			
Primary school	−0.745^**^	0.042	0.101
	(0.306)	(0.229)	(0.228)
Junior high school	−0.39^*^	0.377^**^	0.408^**^
	(0.22)	(0.165)	(0.164)
HJTV	−0.23	0.742^***^	0.761^***^
	(0.222)	(0.166)	(0.165)
College and above	−0.207	0.522^**^	0.539^**^
	(0.322)	(0.241)	(0.24)
Residence type (ref: rural)	1.032^***^	0.087	0.005
	(0.091)	(0.068)	(0.069)
Health insurance (ref: none)	0.51^***^	1.332^***^	1.291^***^
	(0.166)	(0.125)	(0.124)
Smoking (ref: no)	−0.464^***^	−0.073	−0.036
	(0.121)	(0.091)	(0.091)
Drinking (ref: no)	−0.029	0.149	0.151
	(0.148)	(0.111)	(0.11)
Self-assessed health status	0.067^*^	0.477^***^	0.472^***^
	(0.04)	(0.03)	(0.03)
Annual personal income	0.176	0.227	0.213^*^
	(0.157)	(0.117)	(0.117)
Annual medical costs	0.174	−0.004	−0.018
	(0.158)	(0.118)	(0.118)
Constant	0.618^*^	1.371^***^	1.321^***^
	(0.353)	(0.265)	(0.263)
Observations	3,313	3,313	3,313
R-squared	0.076	0.150	0.159
F statistic	18.30	38.66	39.01

HJTV, High School/Junior High School/Technical School/Vocational High School. Robust standard errors in parentheses; number of bootstrap samples for percentile bootstrap confidence intervals is 5,000;

*** p < 0.01,

** p < 0.05,

* p < 0.1.

After confirming the mediating effect of physical exercise, this study proceeded to calculate the total effect, direct effect, and mediating effect (see Table 5). It showed that in the relationship between internet use intensity and quality of life, the mediating effect of physical exercise (0.008) accounted for 10.25% of the total effect (0.078), and 11.94% of the direct effect (0.067). This suggests that 10.25% of the positive effect of internet use intensity on quality of life was mediated by the mediating effect of physical exercise. The 95% confidence intervals do not overlap with 0, which means that they are statistically significant.

**Table 5:** Results of the mediating effect.

	**Effect**	**Boot SE**	**Boot LLCI**	**Boot ULCI**	**Ratio of indirect to total effect**	**Ratio of indirect to direct effect**
Total effect	0.078	0.015	0.049	0.107		
Direct effect	0.067	0.015	0.041	0.099		
Physical exercise	0.008	0.002	0.005	0.013	10.25%	11.94%

Boot SE, Boot LLCI and Boot ULCL is estimated standard error under bias-corrected percentile bootstrap method, and 95% confidence interval lower and 95% confidence interval upper, and Boot LLCI and Boot ULCL do not overlap with zero, number of bootstrap samples for percentile bootstrap confidence intervals is 5,000.

### The moderating effect test of health insurance

The regression results in Table 6 indicate that health insurance positively moderates the relationship between internet use intensity and quality of life. To avoid the effect of multicollinearity, internet use intensity and health insurance were centered separately, and then the interaction (Health Insurance × Internet Use Intensity) was constructed. Internet use intensity, health insurance, and the interaction (Health Insurance × Internet Use Intensity) were placed together in Model 7 for regression analysis. The results showed that the OR of the interaction (Health Insurance × Internet Use Intensity) on quality of life was 1.085, which was statistically significant at the 5% significance level; internet use intensity (OR = 1.082, *p* < 0.01) and health insurance (OR = 3.360, *p* < 0.01) positively affected quality of life, respectively. This suggests that, holding the control variables constant, an increase of 1 h per day in internet use among respondents with chronic diseases is linked with a 36% improvement in quality of life for respondents with health insurance compared to those without health insurance. It implies that the positive relationship between internet use intensity and quality of life is stronger in the sample with health insurance than in the sample without health insurance. These results also indicate that health insurance strengthened the positive relationship between internet use intensity and quality of life. Therefore, hypothesis H3 is supported.

**Table 6:** Regression results of the moderating effect for health insurance (*N* = 3,313).

**Variables**	**Model 7**
Internet use intensity	1.082^***^
	(0.018)
Health insurance (ref: none)	3.360^***^
	(0.415)
Health insurance **×** internet use intensity	1.085^**^
	(0.035)
Gender (ref: female)	1.195^**^
	(0.094)
Age	1.035^***^
	(0.004)
Marriage status (ref: married)	1.269
	(0.233)
Education level (ref: Not educated)	
Primary school	1.016
	(0.214)
Junior high school	1.433^**^
	(0.231)
HJTV	1.910^***^
	(0.323)
College and above	1.485^*^
	(0.343)
Residence type (ref: rural)	1.084
	(0.070)
Smoking (ref: no)	0.925
	(0.084)
Drinking (ref: no)	1.151
	(0.125)
Self-assessed health status	1.576^***^
	(0.048)
Annual personal income	1.275^*^
	(0.181)
Annual medical costs	0.984
	(0.107)
Observations	3,313
Wald chi^2^	467.96
Pseudo *R*^2^	0.037
Log pseudolikelihood	−6741.40

The parameters reported are the odds ratios (OR) of the ordered logistic regression model; robust standard errors in parentheses. HJTV indicates High School/Junior High School/Technical School/Vocational High School.

*** p < 0.01,

** p < 0.05,

* p < 0.1.

### Robustness test

The purpose of the robustness test is to examine the stability of the regression analysis. In other words, the effect of the independent variable on the dependent variable remains stable when the variable measurement is changed, other models are used, or the sample size is changed. At present, there is no standardized robustness test, and subsample regression, reselecting the regression model, making variable substitutions, and changing the sample size are all commonly used robustness tests. We use OLS models to re-test the effect of internet use intensity on quality of life by recoding the independent and dependent variables. Specifically, the 14 question items were formed into one variable using principal component analysis to measure quality of life; the measure of internet use intensity was a categorical variable recoded in five degrees for internet use time. The newly formed internet use intensity and quality of life were then used to conduct regression analyses for the full sample (Model 8), the rural subsample (Model 9), and the urban subsample (Model 10), respectively. The results are shown in Table 7, indicating that the effect of internet use intensity on quality of life is consistent with the results in Table 3, and the results are robust and reliable.

**Table 7:** Results of the relationship between internet use intensity and quality of life (*N* = 3,313).

**Variables**	**Full sample**	**Rural sample**	**Urban sample**
	**Model 8**	**Model 9**	**Model 10**
Internet use intensity	0.035^***^	0.034^***^	0.035^***^
	(0.004)	(0.007)	(0.006)
Gender (ref: female)	0.022^**^	0.019	0.025^*^
	(0.010)	(0.014)	(0.015)
Age	0.004^***^	0.003^***^	0.004^***^
	(0.000)	(0.001)	(0.001)
Marriage status (ref: married)	−0.003	0.004	−0.007
	(0.024)	(0.038)	(0.030)
Education level (ref: Not educated)			
Primary school	0.010	−0.007	0.025
	(0.029)	(0.036)	(0.047)
Junior high school	0.029	0.018	0.035
	(0.022)	(0.035)	(0.029)
HJTV	0.060^***^	0.021	0.078^***^
	(0.023)	(0.039)	(0.029)
College and above	0.029	0.019	0.039
	(0.031)	(0.065)	(0.036)
Residence type (ref: rural)	0.009		
	(0.008)		
Health insurance (ref: none)	0.221^***^	0.232^***^	0.213^***^
	(0.016)	(0.026)	(0.022)
Smoking (ref: no)	−0.017	−0.013	−0.023
	(0.012)	(0.016)	(0.017)
Drinking (ref: no)	0.026^*^	0.008	0.040^*^
	(0.015)	(0.021)	(0.021)
Self-assessed health status	0.036^***^	0.043^***^	0.029^***^
	(0.004)	(0.005)	(0.006)
Annual personal income	0.039^**^	0.062^*^	0.034^**^
	(0.015)	(0.037)	(0.016)
Annual medical costs	−0.007	−0.026	0.001
	(0.015)	(0.023)	(0.021)
Constant	−0.412^***^	−0.419^***^	−0.393^***^
	(0.037)	(0.052)	(0.055)
Observations	3,313	1,582	1,731
R-squared	0.133	0.135	0.127
F statistic	30.68	15.98	16.98

HJTV, High School/Junior High School/Technical School/Vocational High School. Robust standard errors in parentheses;

*** p < 0.01,

** p < 0.05,

* p < 0.1.

## Discussion

Chronic patients' quality of life during the COVID-19 Pandemic should be given attention. Based on the cross-sectional data from the CFPS dataset of wave 2020, the current study validated the underlying mechanism between internet use intensity and quality of life among chronic patients during the COVID-19 Pandemic in China mainland.

This study found that internet use intensity was significantly and positively related to quality of life. In other words, the more frequently individuals use the internet, the higher level of quality of life they experience, and this relationship is strongest in the rural sample, followed by the overall sample and the urban sample. However, this finding is inconsistent with previous studies that more use of the internet could reduce social communication, increase loneliness (56), and enhance sedentary risk (57). A likely explanation is that chronic patients could gain social, emotional, and experiential support from the internet during the lockdown. According to the uses and gratifications theory (58), users seek gratifications from the internet to fill their basic needs. The internet can not only be used for entertainment, work, study, and information seeking, but also as an important intervention tools for people to promote psychological empowerment and rehabilitation (19, 59). This indicates that providing physical activity interventions via the internet will help chronic patients improve their quality of life.

Furthermore, it revealed that physical exercise mediated the association between internet use intensity and quality of life. This finding indicates that people who use the internet more frequently are more likely to participate in physical exercise and report a higher quality of life. It is acknowledged that physical exercise is an effective way to prevent and treat chronic diseases (60, 61). Previous studies have shown that users with higher eHealth literacy are more likely to be physically active, while physical inactivity is a primary cause of most chronic diseases (33, 62, 63). The lockdown policy during the COVID-19 outbreak may lead to sedentary and reduced regular activities, and fewer visits to the hospital to prevent infection, thereby increasing health risks (64, 65). However, people who use the internet more frequently may gain more exercise-related knowledge from online social media platforms, become more accustomed to using social media apps or smart exercise tools for physical exercise, and display physical exercise achievement (66, 67). Therefore, encouraging appropriate physical activity through the internet can help improve the health conditions and quality of life of people with chronic patients.

Besides, this is the first study that revealed the moderating role of health insurance in the relationship between internet use intensity and quality of life. One possible explanation may be that China rolls out online healthcare to tackle a growing number of patients with chronic conditions (68). Patients could use online medical services during the pandemic, and online medical care was covered by Chinese health insurance (69, 70). Prior research revealed that the internet is a major source of health insurance information (71). Another study found that individuals with no medical insurance have had higher disease risk anxiety and lower life satisfaction than those who had at least one insurance (72). The “Internet +Healthcare” policy encourages people to seek medical treatment through the internet. This finding suggests that more online medical insurance policies should be improved to address the medical needs of chronic patients during the lockdown. All in all, this study enlightens us that the internet use intensity can promote quality of life in chronic patients, but its effect is limited, if the internet as an independent factor. There are two mechanisms that play the influence role. One is that physical exercise mediates the positive association between internet use and quality of life, where 10.25% of the effect is mediated by physical exercise. The other is that health insurance enhances the positive relationship between internet use intensity and quality of life.

These findings shed new light on China's healthcare policy. The internet has been used as a substitute for medical suggestions among patients who lack insurance or have difficulty accessing medical treatment (73). In the background of Chinese “Internet +Healthcare” policy, on the one hand, the internet provides online medical services which have improved the efficiency of medical treatment for chronic patients and may improve their quality of life. On the other hand, medical insurance reimbursement through the internet provides convenience for patients with chronic diseases. During the Pandemic, it's necessary to further expand the scope of beneficiaries of the “Internet +Healthcare” policy, such as the application in rural groups, simplify the process of online medical treatment and medical insurance reimbursement, so as to improve the quality of life among chronic patients.

Our study also has some clinical significance. Our findings showed that internet-based physical activity interventions have great potential to improve chronic patients' quality of life. Besides, physical exercise helps to improve the physical function of patients with chronic diseases (74). The internet provides chronic patients with health plans, physical activity testing, goal setting, feedback functions, and self-health management. Generally, internet-based physical activity provides a non-invasive way for chronic disease prevention and treatment (33). In future studies, we should further develop internet-related treatment strategies for patients with chronic diseases clinically.

It is necessary to revise Flanagan's study, which considered quality of life to include five dimensions (physical and material well-being; relationships with other people; social, community and civic activities; personal development and fulfillment; and recreation) (42), but the background in which this idea was developed was in 1978, when the Internet was not shaping human life as profoundly as it does today. It should be taken into consideration that the Internet as a factor is included in the concept of measuring quality of life. This study also responds to Link and Phelan's fundamental-causes theory, which holds that social condition is the underlying cause of health differentiation among people and has a persistent effect on health quality (75). Since the COVID-19 pandemic, the intensity of human internet use has dramatically increased, making the internet a new and important key influence on quality of life among the chronic patients. This extends the explanatory scope of the fundamental-causes theory.

## Limitations

The strength of this study is reflected in several aspects. Firstly, the data we use are from CFPS of wave 2020, which is a national survey covering mainland China that has been ongoing for more than a decade, and ensures the scientific validity and representativeness of the data. Secondly, our data were conducted in the background of the COVID-19 Pandemic in mainland China in 2020, and the latest data were only allowed to be applied for use at the end of 2021, which reflects the latest situation regarding the quality of life of chronic patients. Then, to ensure the reliability of the study findings, we performed robustness tests on the data during the empirical analysis. Finally, we explored the effect mechanism of the relationship between internet use intensity and quality of life among chronic patients by introducing physical exercise (mediating variable) and health insurance (moderating variable).

However, there are some limitations of our study that should be acknowledged. Firstly, a cross-sectional survey has been used so that the causative interpretations could not be determined. Secondly, Due to the limitation of secondary data, we could only refer to the five dimensions of The Flanagan Quality of Life Scale (QOLS) with 14 approximate question items to measure the quality of life among chronic patients, which is still flawed despite validity and reliability tests. Thirdly, the present study focuses only on the internet use intensity and ignores the role of excessive internet use. To further promote chronic patients' quality of life in the future study, we can use the Flanagan Quality of Life Scale to measure quality of life, and focus on the overuse of the internet in chronic patients during the pandemic.

## Conclusions

This paper empirically tested the underlying mechanism of the association between internet use intensity and quality of life among patients with chronic diseases. The findings show that internet use intensity is positively related to the quality of life directly, and indirectly through the mediating role of physical exercise and the moderating role of health insurance. Relevant policies should be developed to improve subjective and objective quality of life during the COVID-19 Pandemic. For patients with chronic diseases, the government can encourage them to use the internet in moderation and maintain physical activity to obtain a higher quality of life. In addition, in the context of universal healthcare, medical insurance should be further covered for each individual to reduce the economic pressure on chronic patients. Thirdly, the “Internet +Healthcare” program should be further promoted so that patients can get better and faster treatment during the COVID-19 Pandemic. Last but not least, even though our results showed that internet use had improved the quality of life of chronic patients, the risks of internet use should not be ignored. The frequency of people's internet usage has increased during the epidemic, so it is necessary to prevent internet dependence from adversely affecting the quality of life.

## Data availability statement

The raw data supporting the conclusions of this article will be made available by the authors, without undue reservation.

## Ethics statement

This study was conducted based on de-identified, publicly available CFPS data and did not interact with any individuals or use identifiable private information. Therefore, the ethics approval was waived. Written informed consent from the [patients/participants OR patients/participants legal guardian/next of kin] was not required to participate in this study in accordance with the national legislation and the institutional requirements.

## Author contributions

YW: framework, model analyses, data curation, writing—original draft, and review and editing. TX: writing—original draft, review and editing, and funding acquisition. JX: supervision. All authors have read and agreed to the published version of the manuscript.

## Conflict of interest

The authors declare that the research was conducted in the absence of any commercial or financial relationships that could be construed as a potential conflict of interest.

## Publisher's note

All claims expressed in this article are solely those of the authors and do not necessarily represent those of their affiliated organizations, or those of the publisher, the editors and the reviewers. Any product that may be evaluated in this article, or claim that may be made by its manufacturer, is not guaranteed or endorsed by the publisher.
